# HIV in Europe and Central Asia: progress in 2018 towards meeting the UNAIDS 90-90-90 targets

**DOI:** 10.2807/1560-7917.ES.2018.23.48.1800622

**Published:** 2018-11-29

**Authors:** Alison E Brown, Rosalie Hayes, Teymur Noori, Yusef Azad, Andrew J. Amato-Gauci, Anastasia Pharris, Valerie C. Delpech, the ECDC Dublin Declaration Monitoring Network

**Affiliations:** 1 Public Health England, London, United Kingdom; 2 Independent Consultant, London, United Kingdom; 3 National AIDS Trust, London, United Kingdom; 4 European Centre for Disease Prevention and Control, Stockholm, Sweden; 5 Independent Consultant, Watipa, London, United Kingdom; 6 The ECDC Dublin Declaration Monitoring group are listed at the end of the article

**Keywords:** HIV, Continuum of HIV Care, Europe and Central Asia, UNAIDS targets

## Abstract

In 2018, 52 of 55 European and Central Asian countries reported data against the UNAIDS 90–90–90 targets. Overall, 80% of people living with HIV (PLHIV) were diagnosed, of whom 64% received treatment and 86% treated were virally suppressed. Subregional outcomes varied: West (87%–91%–93%), Centre (83%–73%–75%) and East (76%–46%–78%). Overall, 43% of all PLHIV were virally suppressed; intensive efforts are needed to meet the 2020 target of 73%.

In 2014, the Joint United National Programme on HIV/AIDS (UNAIDS) established the global 90–90–90 targets. The aim was for 90% of all people living with HIV (PLHIV) to be diagnosed, 90% of those diagnosed to receive antiretroviral treatment (ART) and 90% of those receiving treatment to achieve viral suppression, by 2020 [1]. Here, we describe progress towards the UNAIDS 90–90–90 targets across Europe and Central Asia and discuss whether current performance is sufficient to eliminate HIV transmission.

## The Dublin Declaration on Partnership to Fight HIV/AIDS

Between January and March 2018, the European Centre for Disease Prevention and Control (ECDC) disseminated an online survey to the 55 countries of Europe and Central Asia that comprise the World Health Organization (WHO) European Region, to monitor the implementation of the Dublin Declaration on Partnership to Fight HIV/AIDS [2]. Countries provided estimates of the number and proportion of people within a defined four-stage continuum of care for the most recent year available (Box) [3,4].

**Four-stage continuum of HIV care, global UNAIDS 90-90-90 target and substantive target**

Stage 1: Estimated number of people living with HIV

Stage 2: Number/percentage of stage 1 diagnosed

Stage 3: Number/percentage of stage 2 receiving ART

Stage 4: Number/percentage of stage 3 with viral load < 200 copies/mL (considered as virally suppressed)

• Global UNAIDS 90-90-90 target:

First 90: Number/percentage of all PLHIV who are diagnosed

Second 90: Number/percentage of those diagnosed who are treated

Third 90: Number/percentage of those treated who are virally suppressed

• Substantive target:

Number/percentage of all PLHIV who are virally suppressed.

ART: antiretroviral therapy; PLHIV: people living with HIV.Source: Gourlay et al [4]

Countries also specified the year to which the estimates related, data sources and collection methods and uncertainty bounds for each continuum stage. Where necessary, data were supplemented using Global AIDS Monitoring (GAM) indicators collected by UNAIDS. Data were validated by countries between May and November 2018 and updated accordingly.

## Definitions and analyses

**Figure 1:**
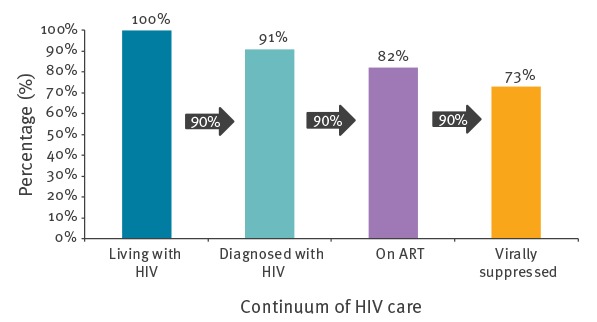
The continuum of HIV care as envisaged by the 90–90–90 UNAIDS targets by 2020

ART: antiretroviral therapy; PLHIV: people living with HIV; UNAIDS: Joint United Nations Programme on HIV/AIDS.

The global 90–90–90 targets are assessed as percentages of each previous stage of the continuum. The ‘substantive target’ is defined as the percentage of all PLHIV who are virally suppressed, making 73% the target (Figure 1). The global targets include countries reporting at least two consecutive stages but the substantive target only includes countries reporting all four stages of the continuum. Data were presented by WHO European subregion (West, Centre and East) which categorises countries in Europe and Central Asia in to three geographic areas by HIV epidemic type [5]. At the (sub)regional level, analyses were undertaken after summing each continuum stage across countries [3]. Data were compared with that previously submitted through the Dublin Declaration survey [3,6,7].

Global 90-90-90 target: each stage of the continuum is presented as a percentage of the previous stage of the continuum, with target of 90%–90%-90%.

Substantive target: each stage of the continuum is presented as a percentage of the total number of people living with HIV, with target of 90%–81%-73%.

Numbers of PLHIV are rounded to the nearest hundred and reported only where available.

Graph is adapted from [1].

## Key findings

In 2018, 52 of 55 countries completed the survey with 34 providing data across all four continuum stages (compared with 29 in 2016) and 42 providing at least two consecutive stages (compared with 41 in 2016). In 2018, progress towards the global 90–90–90 targets in Europe and Central Asia stands at 80%–64%–86%. In the West subregion, 87%–91%–93% was achieved with equivalent figures at 83%–73%–75% and 76%–46%–78% in the Centre and East, respectively. In countries in the European Union/European Economic Area (EU/EEA), which includes countries from each of the subregions, the progress stands at 86%–91%–92%. The number and proportion in each continuum stage are presented in the Table.

**Table:** Progress against the global 90–90–90 targets reported, European and Central Asian countries, 2018 (n = 52)

WHO subregion	Country	Total numbers				Global 90–90–90 Targets			Substantive Target
		All PLHIV	Diagnosed	Treated	Virally suppressed	% of PLHIV who are diagnosed	% of diagnosed PLHIV who are receiving ART	% receiving ART who are virally suppressed	% of PLHIV who are virally suppressed
						2020 target: 90%	2020 target: 90%	2020 target: 90%	2020 target: 73%
West (n=23 countries)	Andorra	NA	68	68	NA	NA	100	NA	NA
	Austria	7,079	6,537	6,145	5,223	92	94	85	74
	Belgium	18,758	15,885	13,763	12,759	85	87	93	68
	Denmark	6,000	5,500	5,300	5,200	92	96	98	87
	Finland	3,880	3,401	NA	NA	88	NA	NA	NA
	France	156,600	132,400	120,700	116,600	85	91	97	74
	Germany	86,100	74,800	68,800	65,500	87	92	95	76
	Greece	16,665	13,866	9,594	NA	83	69	NA	NA
	Iceland	NA	NA	NA	NA	NA	NA	NA	NA
	Ireland	7,205	6,276	5,227	4,986	87	83	95	69
	Israel	8,039	7,448	5,087	NA	93	68	NA	NA
	Italy	130,000	114,400	100,000	87,000	88	87	87	67
	Liechtenstein	NA	NA	NA	NA	NA	NA	NA	NA
	Luxembourg	1,081	919	812	751	85	88	92	69
	Malta	453	340	340	298	75	100	88	66
	Monaco	47	47	47	47	100	100	100	100
	The Netherlands	22,900	20,264	18,599	17,580	88	92	95	77
	Norway	NA	NA	NA	NA	NA	NA	NA	NA
	Portugal	38,959	35,709	31,000	28,007	92	87	90	72
	Spain	146,000	120,000	116,408	103,000	82	97	88	71
	Sweden	8,320	7,489	7,261	6,930	90	97	95	83
	Switzerland	16,600	15,000	14,400	13,900	90	96	97	84
	United Kingdom	101,400	91,987	88,089	85,446	91	96	97	84
	**West total^a^**	**776,086**	**672,336**	**611,640**	**553,227**	**87**	**91**	**93**	**74**
Centre (n=15 countries)	Albania	1,300	891	568	312	69	64	55	24
	Bulgaria	2,862	2,410	1,198	689	84	50	58	24
	Croatia	1,533	1,077	919	822	70	85	89	54
	Cyprus	NA	NA	NA	NA	NA	NA	NA	NA
	Czech Republic	3,230	2,533	1,800	1,660	78	71	92	51
	Hungary	NA	NA	NA	NA	NA	NA	NA	NA
	Kosovo*	NA	NA	NA	15	NA	NA	NA	NA
	The former Yugoslav Republic of Macedonia,	383	246	198	191	64	80	96	50
	Montenegro	437	201	140	121	46	70	86	28
	Poland	NA	NA	NA	NA	NA	NA	NA	NA
	Romania	17,000	15,009	11,570	8,409	88	77	73	49
	Serbia	2,700	2,441	1,724	NA	90	71	NA	NA
	Slovakia	995	756	540	NA	76	71	NA	NA
	Slovenia	987	670	533	530	68	80	99	54
	Turkey	NA	NA	NA	NA	NA	NA	NA	NA
	**Centre total^a^**	**31,427**	**26,234**	**19,190**	**12,749**	**83**	**73**	**75**	**46**
East (n=14 countries)	Armenia	3,400	2,265	1,530	1,304	67	68	85	38
	Azerbaijan	8,003	5,661	4,207	1,778	71	74	42	22
	Belarus	26,120	19,231	11,242	7,253	74	58	65	28
	Estonia	7,900	NA	4,109	NA	NA	NA	NA	NA
	Georgia	10,500	5,090	4,144	3,383	48	81	82	32
	Kazakhstan	26,000	20,841	11,482	6,338	80	55	55	24
	Kyrgyzstan	8,500	5,805	3,237	1,995	68	56	62	23
	Latvia	NA	NA	NA	NA	NA	NA	NA	NA
	Lithuania	2,761	2,601	780	609	94	30	78	22
	Moldova	15,132	11,887	5,162	3,324	79	43	64	22
	Russia	998,525	808,823	319,613	27,1671	81	40	85	27
	Tajikistan	15,000	7516	4,942	NA	50	66	NA	NA
	Ukraine	244,000	136,378	98,237	57,010	56	72	58	23
	Uzbekistan	NA	21,364	20,281	17,530	NA	95	86	NA
	**East total^a^**	**1,365,841**	**1,047,462**	**488,966**	**372,195**	**76**	**46**	**78**	**26**
	**Total^a^**	**2,173,354**	**1,746,032**	**1,119,796**	**938,171**	**80**	**64**	**86**	**43**

ART: antiretroviral therapy; NA: Not available; PLHIV: people living with HIV; WHO: World Health Organization.

^a^ Totals reflect sum of values presented. However only countries with data for consecutive stages of the continuum are included in the global 90–90–90 target outcomes and only countries with all four elements are included in substantive target outcome.

* This designation is without prejudice to positions on status, and is in line with UNSC 1244 and the ICJ Opinion on the Kosovo Declaration of Independence.

**Figure 2:**
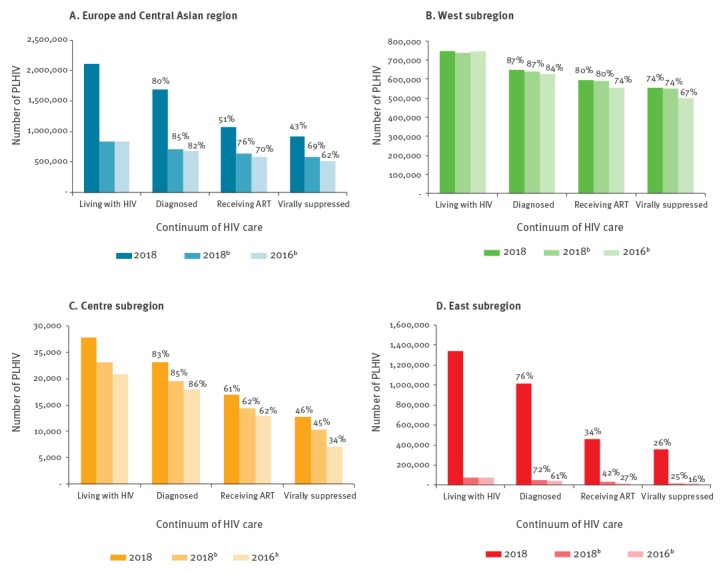
Continuum of HIV care (substantive target), overall and by WHO subregion, 2018, and comparison between 2018 and 2016^a,b^

ART: antiretroviral therapy; PLHIV: people living with HIV; WHO: World Health Organization.

^a^ Countries reporting data for both years included: West: Austria, Belgium, Denmark, France, Germany, Italy, Luxembourg, Malta, the Netherlands, Portugal, Spain, Sweden, Switzerland and the United Kingdom; Centre: Albania, Bulgaria, Croatia, Montenegro, Romania; East: Armenia, Azerbaijan, Georgia, Kazakhstan, Kyrgyzstan and Moldova.

^b^ Countries providing data for both years.

Overall, 43% (n = 920,600/2,118,200) of all PLHIV were virally suppressed (in the 34 countries providing data on all stages of the continuum) (Figure 2). The substantive target was exceeded in the West subregion with 74% (n = 553,200/747,500) of all PLHIV virally suppressed compared with 46% (n = 12,700/27,700) and 26% (n = 354,700/1,342,900) in the Centre and East respectively. In the 20 countries of the EU/EEA that provided data, 73% (n = 552,000/759,200) of all PLHIV were virally supressed.

For countries that provided data for both years (n = 25), performance can be compared between 2016 and 2018 (Figure 2); outcomes improved from 82%–85%–89% to 85%–89%–91% for the global targets, respectively. Overall 69% of all PLHIV were virally suppressed in 2018 compared with 62% in 2016. Improvements in the substantive targets were observed in all subregions (the 1% decline in percentage diagnosed in the Centre subregion is likely due to a revision of the estimate of undiagnosed PLHIV). Viral suppression among all PLHIV was 74% (West), 45% (Centre) and 25% (East) in 2018, compared with 67%, 34% and 16% in 2016, respectively.

**Figure 3:**
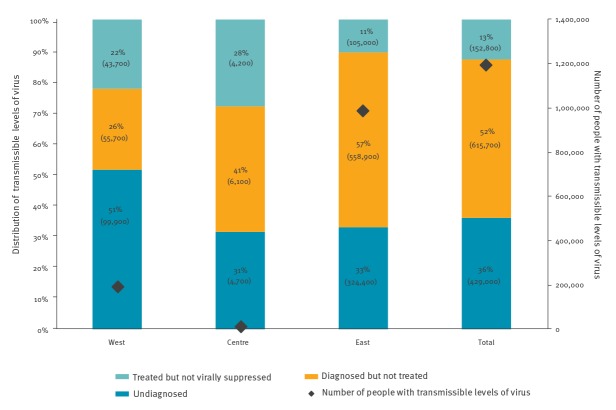
Distribution of people with transmissible levels of virus, by WHO subregion^a^, and Europe and Central Asia overall, 2018

The number of people with transmissible levels of virus can be calculated by adding the number of PLHIV estimated to be undiagnosed, diagnosed but untreated and treated but not virally suppressed (for countries providing all four stages. An estimated 57% (1.2 million/2.1 million) PLHIV are presumed to have transmissible levels of virus in 2018. Of which, an estimated 36% were undiagnosed, 51% were diagnosed but untreated and 13% were treated but had unsuppressed viral load (Figure 3). By subregion, 16% of people with presumed transmissible levels of virus lived in the West, 1% in the Centre and 83% in the East. Excluding Russia (which constituted 60% of all PLHIV with transmissible levels of virus in the entire Region) the figures become 41%, 3% and 56%, respectively.

WHO: World Health Organization.

^a^ Countries reporting all four stages of the continuum.

## Discussion

There has been substantial progress towards achieving the global UNAIDS targets across Europe and Central Asia and improvements have been recognised across all three WHO subregions. Despite this, only two-fifths of all PLHIV across the Region are estimated to be virally suppressed in 2018. While the West subregion exceeded the 73% substantive target, only around a half (Centre) and a quarter (East) of PLHIV are virally suppressed. The substantial progress between 2016 and 2018 is indicative of the improvements that can be made on a Regional scale in a short amount of time; this should provide reassurance and incentive to other global Regions that still have much work in order to meet the 90–90–90 ambition [8].

Each country should assess its progress against 90–90–90 targets and compare outcomes against Regional performance to drive further improvement. Only 34 of 55 countries provided data on all four continuum stages. Improved data availability is crucial to better monitor the effectiveness of their public health response to HIV. Countries that are unable to monitor the continuum of care need support to develop the infrastructure and data flows to enable them to do so.

Improvements in ART access are likely to have positively influenced the outcomes. Four countries recommended ART initiation regardless of CD4^+^ cell count (‘test and treat’) in 2014, 16 in 2016 and 14 in 2018. Treatment coverage among the diagnosed population was 89% for countries implementing ‘test and treat’ in 2014, 93% in 2016 and 46% in 2018. This, together with the significant variation in outcomes across countries and subregions (including those with similar contexts and epidemics) demonstrates the powerful impact of policy implementation. The substantial drop-off between the percentages diagnosed and treated in the East subregion is concerning since it enables preventable deaths, serious illness and onward transmission.

Characterising the population living with transmissible virus provides new insight into the scale and focus of prevention efforts. Almost two-thirds of the 1.2 million people with transmissible virus across the Region are diagnosed. This indicates that the biggest public health impact could be achieved through rapid and sustained scale up of treatment, particularly in the Centre and East subregions. Across the Region, the large undiagnosed population can be addressed by diversifying and augmenting policies offering HIV tests: for indicator conditions, during screenings for other sexually transmitted infections, in community-based settings, as self/home-testing and for partner notification. New European guidance on setting-based approaches for HIV and hepatitis testing can help countries implement more effective testing programmes [9].

It is unclear whether current progress has impacted on HIV transmission. Recent modelling suggests a viral suppression rate of 90% among all PLHIV must be reached to reduce incidence [10]. This indicates intensive efforts are required before transmission begins to fall. However, the West subregion, which has met the substantive target, has provided a favourable context in which reductions in HIV transmission are apparent among gay and bisexual men in some settings [11-14]. It is likely further declines will be observed when pre-exposure prophylaxis (PrEP) is fully implemented to those most at need.

The 90–90–90 targets remain a powerful tool to assess progress towards HIV elimination and drive standards in care for PLHIV. However, they cannot provide a comprehensive picture of the public health response to HIV. While testing and treatment access are a clear focus, deaths and key interventions such as condom use, PrEP and health promotion are excluded. Results are limited in their representativeness, since only 34 countries provided information for all four stages of the continuum with only 25 providing data for both 2016 and 2018. Furthermore, while continuum methods have been defined, in practice, variations in data availability, sources, timeframes and analysis (such as ability to account for deaths and loss-to-follow-ups) limit direct comparisons. Stage 1 of the continuum is, by necessity, an estimation. It is the part of the continuum that is simultaneously most vulnerable to uncertainty and the most critical since it sets the denominator upon which the 90–90–90 targets are calculated [4]. The estimated number of PLHIV (including number with transmissible virus) underrepresents the true Regional situation since they are presented only where countries report data.

A major limitation is that percentage values mask absolute numbers of PLHIV. We recommend that assessment of the 90–90–90 targets must incorporate the number of PLHIV. The analysis of the estimated number of people living with transmissible levels of virus provides further insights. For instance, the high proportion of people with transmissible virus is exacerbated by the large size of the Russian and Ukrainian epidemic. Furthermore, in the United Kingdom, France and Germany, the proportion of people with transmissible HIV is low but masks significant absolute numbers, which impedes efforts to reduce HIV incidence. It is also important to ascertain outcomes for key populations which are known to experience stark inequalities within and between countries [15].

It is crucial not to rest content with meeting the 90–90–90 targets. Each ‘last 10 percent’ includes people especially marginalised from healthcare services. Beyond the 90–90–90 ambition, intensive efforts in policy and service implementation are vital if the ultimate aim of ‘getting to zero’ is to become reality.

## Notes

*This designation is without prejudice to positions on status, and is in line with UNSC 1244 and the ICJ Opinion on the Kosovo Declaration of Independence.

## Notes

**ECDC Dublin Declaration Monitoring Network:** Roland Bani (Albania), Jennifer Fernández Garcia (Andorra), Samvel Grigoryan, Arshak Papoyan, Trdat Grigoryan (Armenia), Irene Kaszoni-Rueckerl, Bernhard Benka (Austria), Esmira Almammadova (Azerbaijan), Inna Karaban, Anna Rusanovich, Svetlana Sergeenko (Belarus), Daniel Reynders, Andre Sasse, Dominique Van Beckhoven (Belgium), Tonka Varleva, Mariya Zamfirova (Bulgaria), Josip Begovac, Tatjana Nemeth-Blazic, Zoran Dominkovic (Croatia), Linos Hadjihannas, Anna Demetriou (Cyprus), Veronika Mičulková, Marek Malý (Czech Republic), Jan Fouchard, Susan Cowan (Denmark), Kristi Rüütel, Tiina Drell (Estonia), Henrikki Brummer-Korvenkontio, Kirsi Liitsola (Finland), Jean-Christophe Comboroure. Florence Lot (France), Ketevan Stvilia, Nikoloz Chkhartishvili (Georgia), Gesa Kupfer, Ulrich Marcus, (Germany), Vasilios Raftopoulos, Sotirios Tsiodras, Dimitra Parasekva (Greece), Katalin Szalay, Maria Dudas (Hungary), Guðrún Sigmundsdóttir (Iceland), Caroline Hurley, Derval Igoe, Kate O’Donnell (Ireland), Daniel Chemtob, Yana Levin (Israel), Anna Caraglia, Francesco Maraglino, Barbara Suligoi, Lella Cosmaro (Italy), Bayserkin Baurzhan Satzhanovich, Irina Ivanova Petrenko (Kazakhstan), Laura Shehu, Pashk Buzhala (Kosovo*), Ulan Kadyrbekov, Aybek Bekbolotov (Kyrgyzstan), Šarlote Konova, Liva Gramatina (Latvia), Irma Caplinskiene (Lithuania), Andrea Leibold, Marina Jamnicki Abegg (Liechtenstein), Patrick Hoffmann, Carole Devaux, Pierre Weicherding (Luxembourg), Jackie Maistre Melillo, Tanya Melillo (Malta), Iurie Climasevschi, Igor Condrat (Moldova), Dominique De Furst (Monaco), Aleksandra Marjanovic, Alma Cicic (Montenegro), Silke David, Ashna Nakched, Eline Op De Coul, Ard van Sighem (Netherlands), Arild Johan Myrberg, Karl Kristian Bekeng (Norway), Iwona Wawer, Piotr Wysocki (Poland), Isabel Aldir, Teresa Melo, Helena Cortes Martins (Portugal), Mariana Mardarescu, Adrian Streinu-Cercel (Romania), Danijela Simic, Sladjana Baros (Serbia), Jan Mikas, Peter Truska (Slovakia), Irena Klavs (Slovenia), Olivia Castillo, Begoña Rodríguez Ortiz de Salazar, Asuncion Diaz (Spain), Maria Axelsson, Gabriella Hok (Sweden), Stefan Enggist, Axel J. Schmidt (Switzerland), Sayfuddin Karimov, Dilshod Sayburhonov (Tajikistan), Emel Özdemir Şahin, Ayla Aydin (Turkey), Valerie Delpech, Peter Kirwan, Alison Brown, Sara Croxford (United Kingdom), Ihor Kuzin, Violeta Martsynovska, Olga Varetska (Ukraine), Gulufar Normuratova, Nurmat Atabekov (Uzbekistan)
